# Fluid management methods for severely burned patients: a narrative review

**DOI:** 10.1007/s10877-025-01365-0

**Published:** 2025-10-16

**Authors:** Yi Yao, Tianzhen Hua, Yucong Li, Meiqing Zhang, Wei Liu

**Affiliations:** 1 Senior Department of Burns and Plastic Surgery, The Fourth Medical Center of Chinese PLA General Hospital, 51 Fucheng Road, Haidian District, Beijing, 100048 China; 2 Chinese PLA Medical School, 28 Fuxing Road, Haidian District, Beijing, 100853 China

**Keywords:** Burn shock, Fluid management, Fluid responsiveness, Fluid tolerance, PiCCO, Critical care ultrasonography

## Abstract

Burn shock is a major early complication in the treatment of severely burned patients, and precise and timely fluid management is essential for survival. Traditional clinical indicators such as urine output, blood pressure, central venous pressure (CVP), and blood lactate are commonly used, but each has significant limitations. Invasive hemodynamic monitoring technologies, such as Pulmonary Artery Catheterization (PAC) and Pulse Contour Cardiac Output (PiCCO), have improved the accuracy of fluid assessment, but carry risks of infection and procedural complications and require experienced clinical interpretation within the context of the patient’s overall condition. Non-invasive ultrasound-based methods, including critical care ultrasonography and the Venous Excess Ultrasound Score (VExUS), are emerging as promising alternatives, particularly in resource-limited settings. This review summarizes current methods for fluid management in severely burned patients, with a focus on the concepts of fluid responsiveness and fluid tolerance, and provides recommendations for clinical practice.

## Introduction

Severe burns (typically defined as those affecting > 20% of total body surface area (TBSA)) [1] are associated with a high risk of shock, which, along with inhalation injury and infection [2], is a leading cause of mortality in this population. Burn shock usually occurs within the first 48 h after injury and is classified as distributive shock. Its pathophysiology involves massive fluid shifts due to increased capillary permeability, peripheral vasodilation caused by tissue necrosis and inflammatory cytokine release, as well as early myocardial depression. These processes collectively lead to both absolute hypovolemia (from fluid extravasation) and relative hypovolemia (due to vasodilation and impaired cardiac function), clinically presenting as systemic hypotension and poor peripheral perfusion. Without intervention, this condition can progress to disseminated intravascular coagulation (DIC) and multiple organ failure [1, 3, 4]. Effective fluid management is therefore critical to navigate the fine line between under- and over-resuscitation. Inadequate resuscitation (under-resuscitation) perpetuates organ hypoperfusion and hypoxia, leading to acute kidney injury and multiple organ failure. Conversely, over-resuscitation, often associated with the phenomenon of “fluid creep,” is equally detrimental and can result in pulmonary edema, extremity and abdominal compartment syndromes requiring emergent intervention, and dilutional coagulopathy [1].

Two key concepts guide modern fluid therapy: fluid responsiveness and fluid tolerance. Fluid responsiveness is defined as the ability of stroke volume(SV) or cardiac output(CO) to increase by typically ≥ 10–15% in response to a fluid challenge (e.g., a rapid bolus of 250–500 mL crystalloids) or a dynamic preload assessment maneuver. Its assessment aims to identify patients who may benefit from additional fluids to optimize intravascular volume and maximize oxygen delivery, while avoiding unnecessary or harmful fluid administration [5]. Fluid tolerance refers to the patient’s ability to receive additional fluids without developing signs of fluid overload or organ dysfunction. Assessing both fluid responsiveness and fluid tolerance is essential for individualized and safe fluid management in burn shock resuscitation.

## Methods for assessing fluid responsiveness and fluid tolerance

### Fluid responsiveness

The assessment of fluid responsiveness is crucial to avoid both under-resuscitation and fluid overload. Methods to assess fluid responsiveness include:

The fluid challenge [6] is a commonly used method for assessing fluid responsiveness in clinical practice. It quickly evaluates fluid responsiveness by observing changes in SV or CO after the rapid infusion of a certain volume of fluid over a short period. However, it inherently carries the risk of iatrogenic fluid overload, particularly in patients who are not fluid-responsive. This risk underscores the importance of using alternative predictive dynamic tests whenever possible before committing to a fluid bolus.

Passive Leg Raising (PLR) [7] is performed by elevating the patient’s lower limbs to about 45°, increasing venous return and temporarily boosting cardiac preload, which hemodynamically mimics a fluid bolus. When assessed by changes in cardiac output (rather than arterial pulse pressure), PLR exhibits excellent predictive accuracy for fluid responsiveness. Its major advantages include being a reversible “virtual” fluid challenge, avoiding the risks of actual fluid administration, and remaining reliable in patients with spontaneous breathing, arrhythmias, or low tidal volume ventilation, where dynamic indices are often unusable. However, its key limitation is that it must be evaluated using a reliable cardiac output monitoring device; reliance on pulse pressure changes significantly reduces its sensitivity. It may also be less reliable in patients with intracranial or intra-abdominal hypertension.

The EEO-test [8] involves interrupting ventilation at end-expiration for 15 s in mechanically ventilated patients. It induces a transient increase in venous return, simulating a fluid challenge. The test shows high diagnostic accuracy with a ~ 5% increase in CO indicating responsiveness. Its advantages include broad applicability across different ventilation settings and compatibility with various CO monitoring devices. Limitations include the need for full mechanical ventilation without spontaneous efforts and precise CO measurement.

The PEEP-test [9] assesses fluid responsiveness by transiently reducing positive end-expiratory pressure (PEEP) from a high level (≥ 10 cmH_2_O) to a low level (5 cmH_2_O) for one minute. This reduction decreases intrathoracic pressure, enhancing venous return and cardiac preload. Simultaneously, it lowers transpulmonary pressure, reducing right ventricular afterload. It demonstrates high predictive value and offers a rapid, non-invasive alternative to fluid challenge, particularly useful when PLR is unreliable. However, it is only applicable in mechanically ventilated patients without spontaneous breathing and with high baseline PEEP, requires advanced hemodynamic monitoring, and carries a risk of transient oxygen desaturation, making it unsuitable for those with acute cor pulmonale or severe hypoxemia.

Not all methods require actual fluid administration, and the choice of method depends on the clinical context and available monitoring tools [10].

### Fluid tolerance

Fluid tolerance is defined as the ability of a patient to receive additional fluids without developing signs of fluid overload, such as pulmonary edema or venous congestion. Assessment of fluid tolerance is essential to prevent complications associated with excessive fluid administration. Common methods include: The extravascular lung water index (EVLWI) measured by transpulmonary thermodilution enables identification of pulmonary fluid accumulation; however, its evidence base for prospectively predicting fluid tolerance is limited [11]. A rising CVP, especially in the context of stable or declining cardiac output, suggests limited fluid tolerance and increased risk of venous congestion or organ dysfunction [12]. Bedside ultrasound can reveal signs of fluid intolerance, such as the development of B-lines in lung ultrasound (which represent established pulmonary edema rather than an early sign) or the dilatation and poor collapsibility of major veins, especially the inferior vena cava (IVC) (though this assessment is challenging in ventilated patients); these findings may suggest impaired ability to handle more fluids even before clinical symptoms develop [13, 14].

## Traditional fluid management indicators

Traditional clinical indicators for fluid management include urine output, blood pressure, and CVP. Urine output is a dynamic and widely used indicator for fluid resuscitation in severely burned patients, but it is a dynamic marker of kidney perfusion that indirectly reflects overall organ perfusion, rather than a direct indicator of volume status. However, it is influenced by various factors such as cardiac function, renal function, and osmotic pressure, which may prevent it from accurately reflecting volume status in certain situations. Moreover, using urine output to guide burn shock treatment may be delayed and needs to be combined with other shock parameters for better guidance in fluid resuscitation [15]. Blood pressure can somewhat reflect the volume status of severely burned patients, relying solely on blood pressure to guide volume assessment is problematic, as it depends on stroke volume—which is influenced by preload, contractility, and afterload—and on systemic vascular resistance, which is inherently variable [16].

### Central venous pressure (CVP)

CVP is a hemodynamic indicator that has been clinically applied for a long time primarily to assess right atrial pressure. While CVP is easy to measure and can provide useful information about right heart preload and the risk of venous congestion, its value as a predictor of fluid responsiveness is limited [17, 18]. CVP is influenced by multiple factors—including intrathoracic and abdominal pressures—and studies have shown that it should not be used alone to estimate blood volume or guide fluid administration. Instead, CVP is best used as a safety parameter: persistently high CVP values are associated with an increased risk of acute kidney injury and other complications [19]. CVP has a very limited role, perhaps in assessing fluid challenge response [20]: for instance, a significant rise in CVP without a concomitant increase in stroke volume may indicate limited ventricular capacity to accept increased preload. When CVP is used as a target value in circulatory failure, it may help most patients reach a satisfactory hemodynamic state but risks subjecting some patients to unnecessary fluid administration, while others may remain under-resuscitated. In those without obvious signs of hypoperfusion, targeting CVP to population-based values is not recommended, as this approach can lead to fluid overload [21].

### Blood lactate

In addition to macrocirculatory indicators, microcirculatory monitoring is also essential. Solely relying on macrocirculatory parameters such as urine output, blood pressure, and CVP may result in situations where these indicators appear normal, but microcirculatory perfusion and tissue oxygenation remain inadequate—a phenomenon known as “macrocirculation-microcirculation decoupling.” Blood lactate is a crucial marker of microcirculatory metabolism and tissue hypoperfusion, and its prognostic value in severely burned patients has been increasingly recognized. Recent studies have shown that serial blood lactate measurements, rather than isolated values, are more effective in predicting outcomes in severely burned patients [22]. Specifically, a reduction or normalization of lactate levels within 24 h after admission is significantly associated with improved survival, highlighting the importance of monitoring lactate trends during resuscitation [22]. Rather than relying on a single cut-off value, the trend of lactate levels (lactate clearance) in the context of multiple clinical parameters provides a better indication of tissue perfusion and the effectiveness of resuscitation. Persistent hyperlactatemia after initial resuscitation may not always indicate ongoing hypoperfusion or the need for further fluid administration, as lactate normalization is often biphasic and influenced by both flow-dependent and non-flow-dependent mechanisms. Overemphasis on lactate normalization as a resuscitation endpoint can increase the risk of fluid overload and related complications [23].

However, lactate is not the only or necessarily the most dynamic indicator of microcirculatory dysfunction. Different perfusion-related variables normalize at different rates during resuscitation. For example, capillary refill time (CRT) and the central venous-to-arterial carbon dioxide pressure difference (Pcv-aCO_2_) often respond more rapidly to resuscitation than lactate, and can provide valuable real-time information for guiding fluid therapy [23].

### Capillary refill time (CRT)

CRT is a simple, non-invasive bedside test that reflects peripheral perfusion. It is measured by applying pressure to a distal extremity until the skin blanches, then timing how long it takes for color to return after releasing the pressure [24]. A prolonged CRT (commonly >2 s in adults) indicates impaired peripheral perfusion and has been associated with worse outcomes in critically ill patients. CRT responds rapidly to fluid resuscitation and other interventions, often normalizing within the first few hours if tissue perfusion improves, making it a valuable real-time indicator for guiding resuscitation, especially when used in conjunction with other parameters. CRT showed a faster initial improvement, making it more responsive to increasing oxygen delivery. These differences highlight CRT’s sensitivity in early resuscitation compared to lactate, which may take longer to normalize [23, 24].

### The central Venous-to-arterial carbon dioxide pressure difference (Pcv-aCO2)

The central venous-to-arterial carbon dioxide pressure difference (Pcv-aCO_2_) is another important marker of tissue perfusion and oxygen metabolism. Pcv-aCO_2_ has been shown to normalize more quickly than lactate during effective resuscitation, providing early feedback on the adequacy of tissue perfusion. Combined monitoring of Pcv-aCO_2_ and central venous oxygen saturation (ScvO2) can offer a more comprehensive assessment of both oxygen delivery and utilization [25, 26], helping to guide fluid therapy and avoid both under- and over-resuscitation.

### Central venous oxygen saturation (ScvO_2_)

ScvO_2_, the oxygen saturation of hemoglobin in the central venous blood, reflects the balance between global oxygen delivery and consumption. A normal ScvO_2_ value is approximately 70–75%. A low ScvO_2_ suggests inadequate oxygen delivery relative to consumption, often due to low cardiac output, anemia, or hypoxemia, and may indicate the need for interventions to improve oxygen supply. It is distinct from mixed venous oxygen saturation (SvO_2_), which is measured in the pulmonary artery via a PAC and is generally 2–5% lower than ScvO_2_ due to coronary sinus drainage. ScvO_2_ is a simple surrogate for SvO_2_, but they are not entirely equivalent. In stable patients, the difference is minimal (with ScvO_2_ typically being slightly higher), but in critically ill or shock states, the two may diverge significantly. Therefore, ScvO_2_ is better suited as a trend indicator rather than an absolute replacement for the precise target of SvO_2_[27].

## Cardiac output monitoring techniques

The invention of the pulmonary artery catheter in the 1970 s marked the beginning of the era of invasive hemodynamic monitoring [28]. In recent years, cardiac output monitoring technology has advanced rapidly, with the emergence of a series of monitoring technologies represented by PICCO and FloTrac/Vigileo, shifting from invasive to minimally invasive and even non-invasive methods [29]. The application of cardiac output monitoring technology has enabled real-time monitoring of the volume status and fluid responsiveness of shock patients, providing quantitative reference indices for fluid resuscitation therapy.

### Pulmonary artery catheter (PAC)

The Pulmonary Artery Catheter (PAC), also known as the Swan-Ganz catheter, enables direct measurement of pressures at various points along the catheter path, including CVP, right atrial pressure, Pulmonary Artery Pressure (PAP), Pulmonary Artery Wedge Pressure (PAWP), and Pulmonary Capillary Wedge Pressure (PCWP). When combined with techniques such as thermodilution, it can also measure Cardiac Output (CO) and mixed venous oxygen saturation (SvO2), allowing real-time monitoring of both left and right ventricular preload and continuous assessment of right heart function in critically ill patients [30]. In severely burned patients, PAC can dynamically evaluate cardiac volume status and right heart function by monitoring real-time CO values and right atrial pressure, and, together with CVP and PCWP, assess the risk of pulmonary edema, thereby indirectly reflecting fluid tolerance. However, PAC has limited ability to predict fluid responsiveness [31], as CO measurements can be influenced by intracardiac shunts, fluid administration, and other factors. Additionally, long-term catheterization increases the risk of infection, thrombosis, pulmonary infarction, and cardiac arrest, making prolonged or repeated monitoring challenging. Therefore, PAC is currently not recommended as the first-line tool for fluid management in severely burned patients.

### Pulse wave analysis (PWA)

Pulse Wave Analysis (PWA) estimates CO by analyzing arterial blood pressure waveform and can also assess Pulse Pressure Variation (PPV) and Stroke Volume Variation (SVV), which are reliably predictive of fluid responsiveness only in deeply sedated, mechanically ventilated patients with sinus rhythm and a tidal volume of at least 8 mL/kg, as they rely on heart-lung interactions [32, 33].

**Fig. 1:**
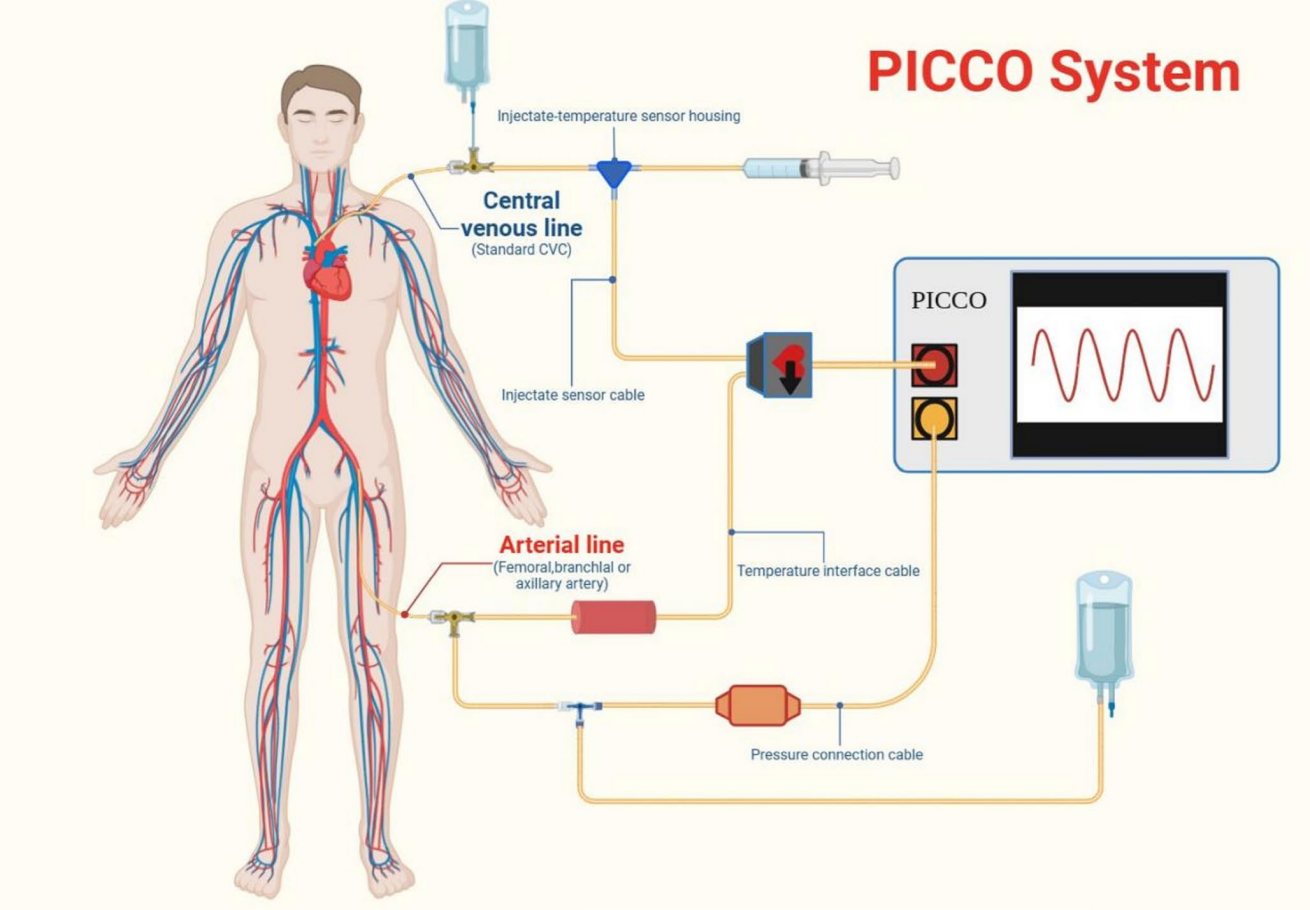
The figure illustrates the key components and operational setup of the PiCCO system, including the integration of arterial line, central venous line, and the monitoring unit. PiCCO combines transpulmonary thermodilution and arterial pulse contour analysis to provide continuous measurements of cardiac output, stroke volume variation, and other volume-related parameters.

The PiCCO system integrates PWA with Transpulmonary Thermodilution (TPTD) technology, calibrating PWA-derived CO with TPTD measurements to provide comprehensive monitoring of hemodynamics, cardiac function, and pulmonary water indices [11, 34]. It directly measures CO via TPTD and arterial pressure. It then calculates parameters such as the Cardiac Index (CI), Systemic Vascular Resistance Index (SVRI), Global End-Diastolic Volume Index (GEDVI), and Intrathoracic Blood Volume Index (ITBI), offering a more accurate reflection of cardiac preload in severely burned patients [35]. Importantly, PiCCO can assess fluid tolerance by monitoring the Extravascular Lung Water Index (EVLWI), which provides a more intuitive and reliable evaluation of pulmonary edema risk compared to PCWP via PAC, though its predictive value for fluid tolerance remains limited. However, the invasive nature of PiCCO still carries risks of infection and cardiovascular responses, and measurement errors may occur in cases of hypothermia, intracardiac shunts, severe valvular regurgitation, aortic aneurysm, or aortic stenosis (Fig. 1).

The FloTrac/Vigileo system estimates real-time CO, CI, SV, and SVV by analyzing peripheral arterial pressure waveforms in conjunction with patient-specific physiological parameters (such as gender, age, body surface area, and vascular compliance) and waveform characteristics [36–38]. Using minimally invasive radial artery catheterization, this system is less traumatic and easier to operate than PAC, making it suitable for guiding early fluid resuscitation in severely burned patients. Through dynamic parameters like SVV, the FloTrac/Vigileo system can assesses fluid responsiveness and supports fluid management, demonstrating predictive accuracy comparable to that of the PiCCOplus system [39]. However, it is currently only applicable to adults, and its accuracy may be compromised in patients with aortic valve regurgitation or severe arrhythmias. Unlike PAC or PiCCO, it does not provide direct indicators of fluid tolerance (such as pulmonary edema risk).

Other PWA technologies include the MostCare system (which uses high-frequency sampling to analyze CO), all of which are invasive. There are also non-invasive systems such as ClearSight and CNAP, which use finger cuffs or intermittent upper arm cuff oscillometry to analyze CO [40, 41]. The PiCCO system combine pulse wave analysis with indicator dilution for external calibration, while the FloTrac/Vigileo, ClearSight, and CNAP systems use biometric, demographic, and hemodynamic data along with arterial waveform characteristics for internal calibration. The MostCare system relies solely on waveform characteristics without internal or external calibration. Although systems with internal calibration reduce the risk of infection associated with invasive monitoring, their measurement accuracy is limited, and their application in fluid management for severely burned patients requires further study.

In summary, technologies such as PAC, PiCCO, and FloTrac/Vigileo each have their advantages and limitations. Dynamic parameters (such as PPV and SVV) are effective for assessing fluid responsiveness, while static parameters (such as PCWP and EVLWI) help determine fluid tolerance. Rational selection and combination of these monitoring methods can optimize fluid management in severely burned patients and reduce the risk of fluid overload and related complications (Table 1).

**Table 1:**
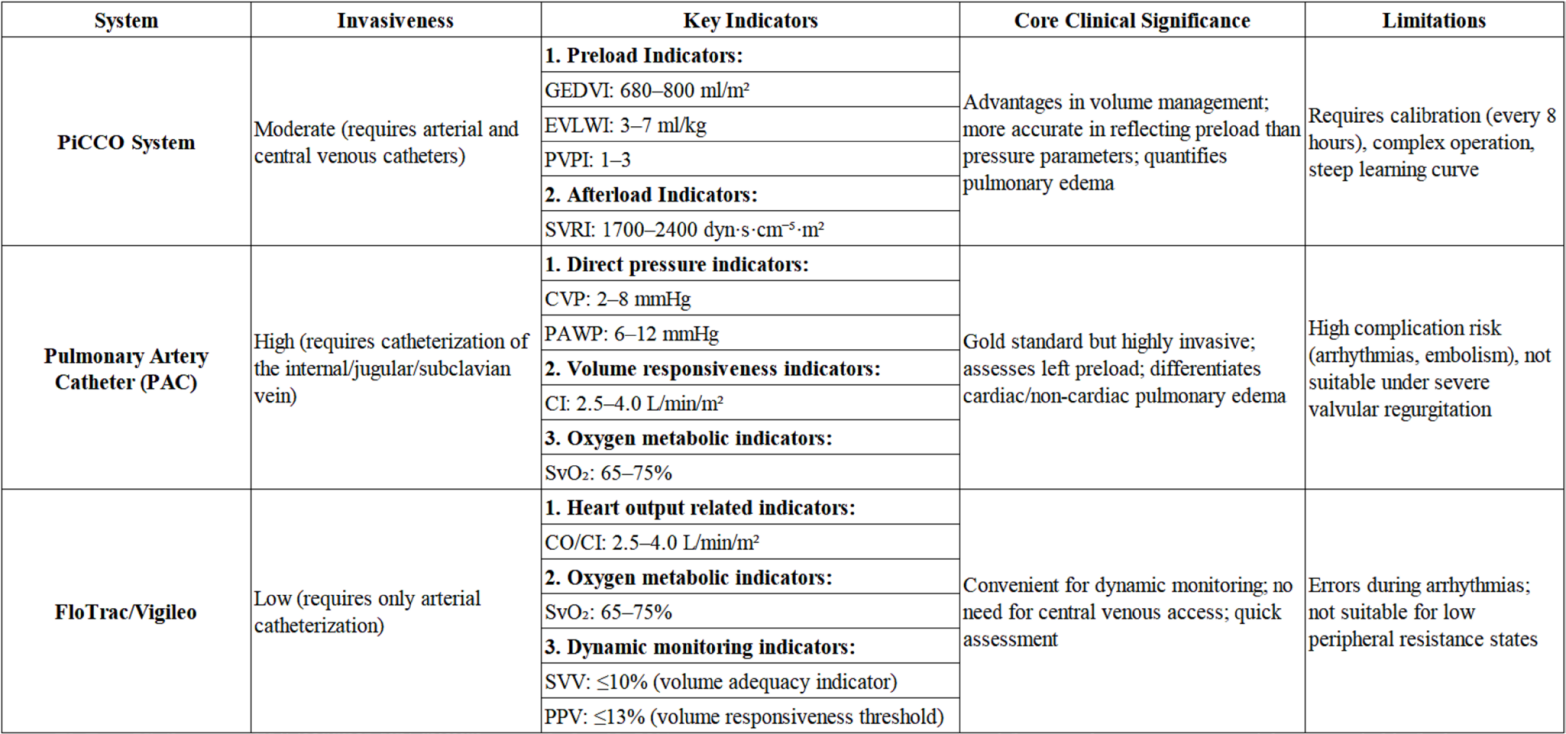
This table compares the core features of three cardiac output monitoring techniques(PiCCO, PAC, and FloTrac/Vigileo), including invasiveness, key indicators, clinical significance, and limitations. The PiCCO system is moderately invasive, focuses on volume-related indicators, is beneficial for precise volume management, but requires regular calibration; the PAC is a highly invasive gold standard that can differentiate between types of pulmonary edema, but has a high risk of complications; FloTrac/Vigileo has low invasiveness, is convenient for dynamic monitoring, but may have errors in conditions such as arrhythmias.

## Ultrasound technology

Ultrasound technology has been widely used for evaluating volume status and fluid responsiveness. Echocardiography can comprehensively assess cardiac preload, contractile function, valve function, and ventricular wall segments. Ultrasound contrast techniques can achieve imaging of organ blood flow and cavities, indirectly inferring the condition of the organs and blood perfusion via Doppler parameters. In terms of fluid management for severely burned patients, methods such as the pulmonary artery catheter, PiCCO, and FloTrac primarily involve invasive arterial blood flow monitoring. Although their effectiveness is more precise compared to traditional indices like urine output and blood pressure, invasive operations and prolonged catheter placements pose infection risks during the treatment of severely burned patients. In contrast, ultrasound technology offers the advantages of being non-invasive, convenient, intuitive, and repeatable. Especially in recent years, the concept of critical care ultrasonography has been emphasized, highlighting the importance of target-oriented dynamic assessment and rapidly developing fluid monitoring in shock patients.

### Critical care ultrasonography (CUS)

Critical Care Ultrasonography refers to the process of dynamic, multi-targeted assessments guided by critical care principles using ultrasound technology, which is problem-oriented for critically ill patients. It allows for rapid analysis of etiologies and is an essential tool for determining the direction of hemodynamic treatments and fine-tuning therapies in critically ill patients. Guided by the concept of critical care ultrasonography, transthoracic and transesophageal ultrasound can be used to assess cardiac pump function; monitoring respiratory variations in the inferior vena cava (ΔIVC) can determine fluid responsiveness; and lung ultrasound can be utilized to assess the degree of pulmonary edema by examining pulmonary B-lines. Critical care ultrasonography shows tremendous potential in evaluating the volume status of severely burned patients.

In fluid management of critically ill patients, guided by critical care principles, transthoracic echocardiography (TTE) and transesophageal echocardiography (TEE) can be utilized to measure CO and SV, as well as the Left Ventricular Outflow Tract Velocity Time Integral (LVOT-VTI) to assess volume status [42]. Volume status can also be evaluated by assessing the diameters of volume-bearing vessels such as the superior and inferior vena cava, and their respiratory variation indices (SVC, IVC) [43, 44], with SVC collapsibility measured via TEE being superior in predicting fluid responsiveness compared to IVC [45]. These measurements, along with indicators like Pcv-aCO2, can better guide fluid resuscitation in patients with septic shock. By measuring the Left Ventricular Stroke Work Index (LVSWI) through TTE, the severity of shock can be assessed, and risk stratification can be performed for patients with different levels of shock [46]. Compared to TEE, TTE is easier to operate and allows for repeated measurements, but its monitoring effectiveness can be affected by factors such as the patient’s physique, thoracic injuries, pulmonary emphysema, bandaging materials, and patient cooperation. TEE, conducted by inserting an ultrasound probe through the esophagus, effectively avoids interference from the chest wall and lungs, providing more accurate and direct monitoring results. It is important to note that TEE is almost exclusively performed by trained cardiologists, cardiac anesthesiologists, or intensivists with specific expertise, and is not typically within the standard skillset of general intensivists or burn surgeons. However, it may cause transient blood pressure changes in patients and poses risks such as arrhythmias and esophageal perforation. Additionally, the discomfort associated with its use limits.

Recent high-quality evidence further underscores the clinical value of point-of-care ultrasound (POCUS) in hemodynamic monitoring and fluid management. POCUS-guided resuscitation in patients with shock can meaningfully influence clinical outcomes. Specifically, POCUS guidance was associated with a probable reduction in 28-day mortality, shorter duration of vasoactive medication use, and a decreased need for renal replacement therapy. While POCUS may not significantly alter the total volume of intravenous fluids or inotropes administered, it likely reduces the number of diagnostic echocardiograms required and improves lactate clearance. These findings highlight the role of POCUS not only in optimizing fluid responsiveness and tolerance assessment but also in improving patient-centered outcomes and resource utilization in critical care settings. However, the impact of operator skill, scan frequency, and timing on these outcomes remains to be fully elucidated [45].

In summary, Critical Care Ultrasonography—especially when applied as point-of-care ultrasound—has become an essential, evidence-based tool for hemodynamic monitoring and fluid management in critically ill and severely burned patients, offering both diagnostic and prognostic benefits that can directly influence clinical decision-making and patient outcomes.

### Lung ultrasound (LUS)

Lung ultrasound (LUS) [47] is an important component of Critical Care Ultrasonography. It assesses pulmonary water content by detecting the number of B-lines produced by multiple reflections of the ultrasound beam at air-fluid interfaces [13]. Studies [14] have shown that using an LUS score for quantitative assessment of B-lines is helpful in evaluating conditions such as atelectasis, pulmonary edema, and pulmonary congestion in critically ill patients. This information is highly valuable in assessing a patient’s volume status and is widely used in fluid management in clinical practice. The combination of LUS and inferior vena cava (IVC) ultrasound can effectively assist in guiding volume management strategies in patients with ARDS [48].

**Fig. 2:**
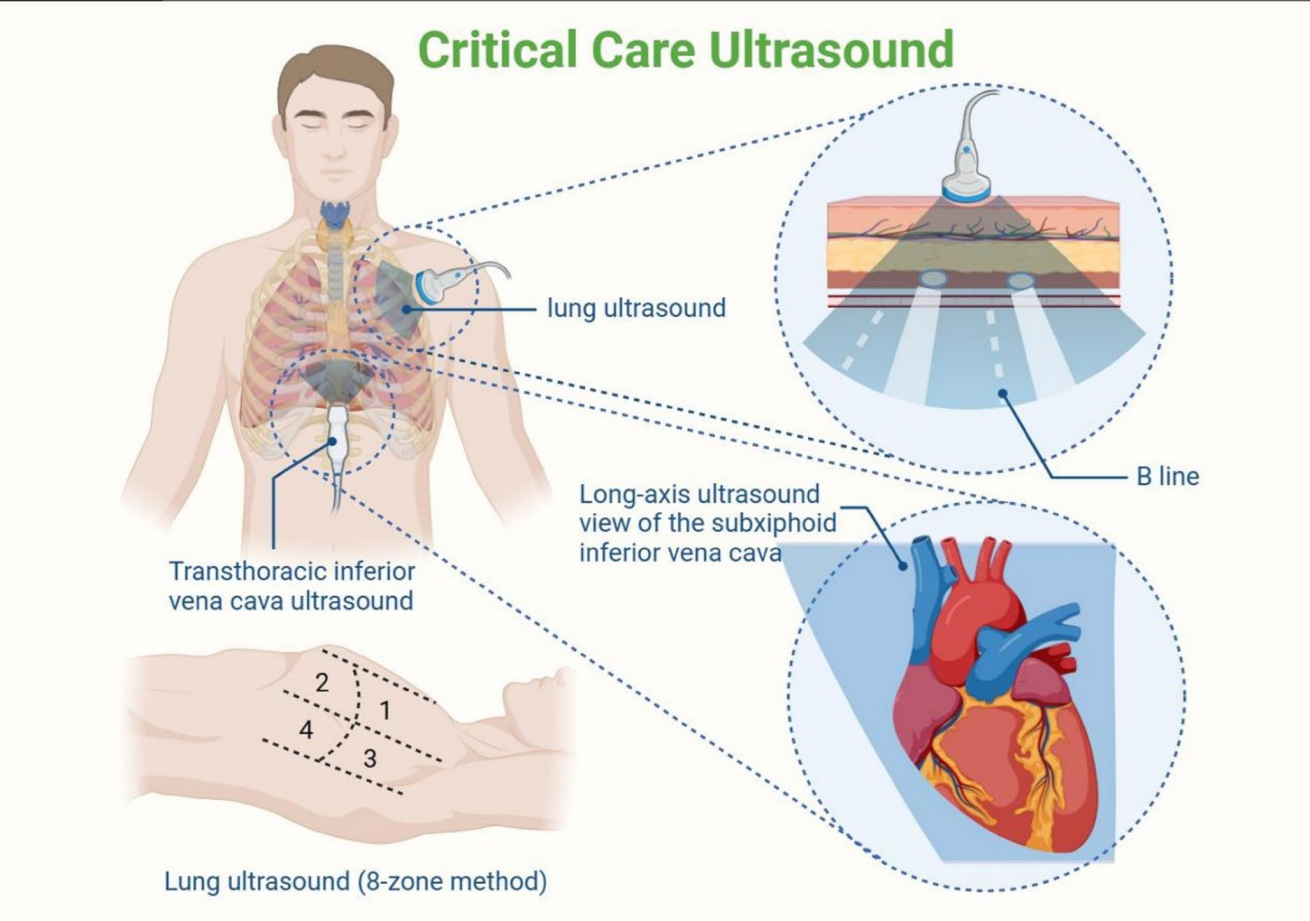
Critical care ultrasonography can be used to assess cardiac pump function; monitoring respiratory variations in the inferior vena cava (ΔIVC) can determine fluid responsiveness; and lung ultrasound can be utilized to assess the degree of pulmonary edema by examining pulmonary B-lines.

The bedside acute lung ultrasound protocol (BLUE protocol) and lung ultrasound-guided shock assessment protocol (FALLS protocol) were developed for rapid diagnosis of acute and severe cardiopulmonary conditions in emergency and ICU settings [49]. Although these two protocols have not yet been specifically applied in severely burned patients, it is noteworthy that when severely burned patients develop complications such as acute lung injury or pulmonary edema, their pathophysiological mechanisms—such as increased capillary permeability and alveolar interstitial fluid extravasation—are similar to those observed in other critically ill patients in the ICU. Thus, the existing BLUE and FALLS protocols may offer valuable references for the rapid diagnosis and volume management of acute pulmonary events in severely burned patients. The future application of these protocols in this population warrants further clinical exploration and research (Fig 2).

### Venous excess ultrasound score (VExUS)

VExUS is a comprehensive ultrasound-based scoring system developed to assess systemic venous congestion. Introduced by Beaubien Souligny and colleagues in 2020, the VExUS system [50] begins with the measurement of the inferior vena cava (IVC) diameter and incorporates Doppler assessments of the hepatic, portal, and renal veins. By combining qualitative and quantitative evaluations of venous congestion, the VExUS score provides a graded assessment of systemic congestion severity. This scoring system has been used to guide targeted fluid removal in cardiorenal syndrome and to optimize fluid management in heart failure, potentially offering a critical threshold for precise fluid resuscitation [51]. Studies have shown that the VExUS score correlates well with changes in right atrial pressure and can effectively predict the development of acute kidney injury [52, 53]. However, its application in patients with severe burns remains limited, partly due to challenges such as extensive skin injury and dressing coverage, which may affect ultrasound image quality.

Despite the widespread use of IVC diameter and its respiratory variability for volume assessment, relying solely on the IVC has notable limitations. An enlarged IVC does not always indicate fluid overload, nor does its dilation quantify the degree of venous congestion in other vital organs such as the lungs, liver, intestines, and kidneys. Therefore, using only the IVC for volume status assessment may lead to incomplete or misleading conclusions, especially in critically ill or severely burned patients.

To address these limitations, recent research has explored the use of alternative veins for fluid management. The axillary vein, in particular, has been proposed as a promising substitute. The ultrasound probe can be optimally aligned with the long axis of the axillary vein, resulting in a larger and more accessible cross-sectional area compared to the subclavian vein [54]. Unlike the internal jugular vein, which exhibits greater variability, or the femoral vein, which shows less, the axillary vein is not affected by intra-abdominal pressure and may provide more reliable volume assessment [55, 56]. This hypothesis suggests that the axillary vein could serve as an effective alternative to the IVC for volume evaluation, especially in situations where IVC imaging is challenging or unreliable.

Evidence supports ultrasound technology as an indispensable tool for the individualized fluid management of severely burned patients [57]. By enabling dynamic, non-invasive assessments of both cardiac function and systemic venous congestion, ultrasonography assists clinicians in evaluating key parameters such as fluid responsiveness and tolerance at the bedside.

## Conclusion and prospects

Precise fluid management remains a cornerstone in the care of severely burned patients, directly impacting resuscitation strategies and clinical outcomes. Traditional indicators such as urine output, blood pressure, and CVP have notable limitations in accurately and promptly reflecting hemodynamic status. Invasive monitoring techniques, including the pulmonary artery catheter (PAC) and pulse contour analysis systems like PiCCO, provide bedside access to a wide range of quantitative hemodynamic parameters. While these systems can be operated by trained clinicians, the data they generate are complex and can be inaccurate in certain clinical scenarios; their meaningful interpretation requires significant expertise to integrate into the overall clinical picture. While the risks of infection and procedural complications must be considered, these techniques remain valuable tools, especially when rapid, accurate, and continuous monitoring is required.

On the other hand, non-invasive and minimally invasive methods—particularly critical care ultrasonography and scoring systems such as the VExUS—are emerging as promising alternatives. These approaches offer the advantages of being repeatable, safe, and capable of providing dynamic, multi-dimensional assessments of fluid responsiveness and tolerance. However, their accuracy and reliability are highly dependent on operator expertise and experience, and their widespread adoption requires ongoing training and standardization.

Ultimately, the choice of monitoring tool should be tailored to the specific clinical context, patient characteristics, and available resources. For example, in the early, unstable phase of burn shock, an invasive system like PiCCO may be preferred for its continuous data and volumetric indices. Once stabilized, a shift to ultrasound-guided management may reduce invasive risks. In resource-limited settings, reliance on sophisticated invasive monitoring may not be feasible, necessitating a greater emphasis on clinical exam, lactate trends, and basic ultrasound. The integration of both invasive and non-invasive methods can provide the most comprehensive and nuanced understanding of a patient’s hemodynamic status, allowing for individualized and adaptive fluid management strategies. Future research should focus on optimizing the combination of these modalities, developing standardized protocols, and validating their effectiveness in diverse patient populations. With continued technological innovation and interdisciplinary collaboration, the field is moving toward more precise, safe, and personalized fluid management for severely burned patients.

## Data Availability

No datasets were generated or analysed during the current study.
